# Managing Type 2 Diabetes Mellitus via the Regulation of Gut Microbiota: A Chinese Medicine Perspective

**DOI:** 10.3390/nu16223935

**Published:** 2024-11-18

**Authors:** Chester Yan Jie Ng, Linda Zhong, Han Seong Ng, Kia Seng Goh, Yan Zhao

**Affiliations:** 1 School of Biological Sciences, Nanyang Technological University, 60 Nanyang Drive, Singapore 637551, Singapore; 2 Singapore General Hospital, Outram Rd., Singapore 169608, Singapore; 3 Academy of Chinese Medicine Singapore, 705 Serangoon Road, Singapore 328127, Singapore; 4 Singapore College of Traditional Chinese Medicine, 640 Lor 4 Toa Payoh, Singapore 319522, Singapore

**Keywords:** type 2 diabetes mellitus, T2DM, Chinese medicine, gut microbiota, gut microbiome

## Abstract

Background: Type 2 Diabetes Mellitus (T2DM) is a metabolic disorder characterized by insulin resistance and inadequate insulin production. Given the increased frequency of T2DM and the health issues it can cause, there is an increasing need to develop alternative T2DM management strategies. One such approach is Chinese Medicine (CM), a complementary therapy widely used in T2DM treatment. Given the emphasis on gut microbiota in current research, studying CM in the treatment of T2DM via gut microbiota modulation could be beneficial. Scope and approach: The use of various CM methods for managing T2DM via gut microbiota modulation is highlighted in this review. Following an introduction of the gut microbiota and its role in T2DM pathogenesis, we will review the potential interactions between gut microbiota and T2DM. Thereafter, we will review various CM treatment modalities that modulate gut microbiota and provide perspectives for future research. Key findings and discussion: In T2DM, Akkermansia, Bifidobacterium, and Firmicutes are examples of gut microbiota commonly imbalanced. Studies have shown that CM therapies can modulate gut microbiota, leading to beneficial effects such as reduced inflammation, improved metabolism, and improved immunity. Among these treatment modalities, Chinese Herbal Medicine and acupuncture are the most well-studied, and several in vivo studies have demonstrated their potential in managing T2DM by modulating gut microbiota. However, the underlying biomolecular mechanisms of actions are not well elucidated, which is a key area for future research. Future studies could also investigate alternate CM therapies such as moxibustion and CM exercises and conduct large-scale clinical trials to validate their effectiveness in treatment.

## 1. Introduction

Type 2 Diabetes Mellitus (T2DM) is a metabolic condition that causes hyperglycemia due to insulin resistance and inadequate insulin production [1]. According to the International Diabetes Federation, around 463 million individuals were diagnosed with diabetes in 2019, and this number is predicted to reach 700 million by 2045, with T2DM accounting for more than 90% of these cases [2]. Changes in food and activity habits, such as consuming more calories and participating in less physical exercise, are leading to a rapid rise in the prevalence of T2DM and growing demands on healthcare resources [3,4]. T2DM has also been identified as a main cause of major health complications such as cardiovascular disease, stroke, and limb amputation [5]. Given its growing prevalence, proper worldwide planning is essential to mitigate the potential health implications of T2DM [6].

Currently, common treatment measures include the use of insulin therapy or pharmacological drugs. Some examples of pharmacotherapy measures include metformin and sulfonylureas [7]. Non-pharmacological options, such as lifestyle interventions, also exist for T2DM patients [3]. However, there are still certain downsides to the standard treatment procedures implemented today. For instance, some drawbacks of insulin therapy include the large number of injections, a lower quality of life, needle bruises, and scarring [8,9]. Similarly, metformin consumption could lead to gastrointestinal adverse effects [10,11,12]. Therefore, individual variability is a crucial component that influences patients’ responses to therapies and, hence, there is an increasing need to source for new forms of T2DM therapy [13].

Recent research has shown that one alternative target of treatment is the gut microbiota [14,15]. In healthy individuals, the gut microbiota mediates physiological processes, thus, maintaining an equilibrium between the body and environment [16]. However, the onset of T2DM may cause gut microbiota dysbiosis, which disrupts one’s internal environment, thus, increasing the chances of opportunistic pathogen proliferation [17]. In recent years, more research groups have begun investigating the impacts of Chinese Medicine (CM) on gut microbiota modulation. Among the treatment modalities of CM, Chinese Herbal Medicine (CHM) has been the most extensively researched in recent years for its gut microbiota regulating effects [18,19]. Furthermore, another CM treatment modality, acupuncture, has been proposed to regulate intestinal inflammation and neurotransmitter secretion. The vagus nerve (VN) is responsible for transmitting changes in gut microbiota and immune activation signals to the brain to establish a neuroregulation pathway [20,21]. Collectively, CM has potential to be utilized in treatment for T2DM.

To the best of our knowledge, past reviews have focused primarily on the anti-diabetic effects of CHM and dietary compounds [18,22,23,24,25]. Hence, our present review aims to complement past findings and provide an overview of the gut microbiota before summarizing its role in T2DM pathogenesis. In addition, we also intend to provide an updated review of CHM and evaluate other different CM treatment modalities for T2DM that modulate gut microbiota. Lastly, we will also present perspectives for future research and development.

## 2. The Gut Microbiota

The gut microbiota includes bacteria, viruses, archaebacteria, and fungus [26]. Amongst the components of the digestive tract, the intestine contains the largest volume of gut microbiota in the human body, with an estimated quantity of 10 trillion colonized bacteria [27]. Its composition and proportion does not remain permanent and may change during one’s lifetime depending on one’s race, age, and diet [28]. Despite gut microbiota composition varying from person to person, cohort studies have shown that the most common gut microbiota still consists primarily of Actinobacteria, Bacteroidetes, Firmicutes, and Proteobacteria [29].

In a physiologically healthy state, the gut microbiota is maintained at a dynamic balance where these bacteria have symbiotic and mutualistic relationship with our bodies [30,31]. The gut microbiota’s primary roles include food digestion, vitamin formation, immune regulation, pathogen growth inhibition, and toxin removal [32,33]. However, a disbalance in the internal gut environment could lead to the onset of metabolic dysfunctions such as T2DM [34,35]. In a previous review conducted by Gurung et al., it was found that Akkermansia, Bacteroides, Bifidobacterium, Faecalibacterium, and Roseburia were negatively associated with T2DM, while Blautia, Fusobacterium, and Ruminococcus were positively associated with T2DM [36]. Amongst them, Bacteroides and Bifidobacterium were also found to be the most common beneficial genera reported in studies of T2DM. Changes to microbial diversity such as an increased Firmicutes/Bacteroidetes ratio also affects insulin resistance and energy metabolism in T2DM pathogenesis [37].

## 3. Potential Mechanisms of Interaction Between Gut Microbiota and T2DM

**Figure 1:**
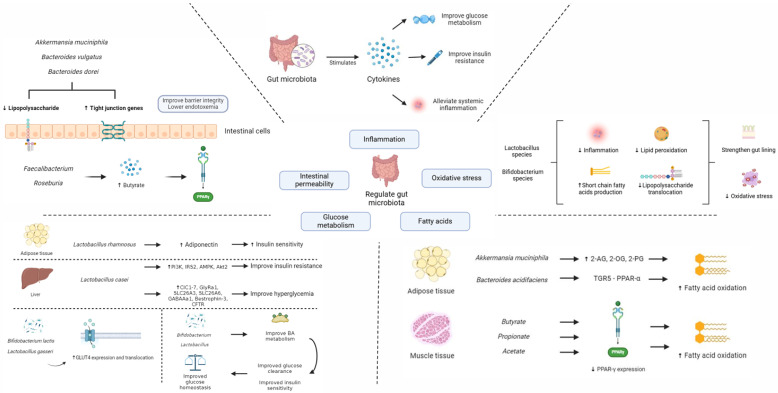
Summary of potential interactions between the gut microbiota and T2DM.

In T2DM, gut microbiota plays an important role in modulating inflammation, interacting with dietary constituents, regulating gut permeability, glucose and lipid metabolism, insulin sensitivity, and maintaining homeostasis [38,39,40,41]. In the sections below, we summarized the potential mechanisms of gut microbiota modulation on the pathogenesis of T2DM. A summary of the potential interactions between the gut microbiota and T2DM is shown in Figure 1 below.

### 3.1. Intestinal Permeability

Increased intestinal permeability is a pathological feature of T2DM, which results in more gut microbial products entering the blood, leading to metabolic endotoxemia [42]. This increase in intestinal permeability, known as “leaky gut”, causes systemic inflammation, which can aggravate insulin resistance and lead to diabetic complications such as neuropathy and retinopathy [43,44]. Tight junction proteins (TJP) are a class of proteins crucial to improving intestinal permeability and studies have shown that administration of live *Bacteroides vulgatus* and *Bacteroides dorei* could improve regulation of tight junction genes, lipopolysaccharide (LPS) production, and endotoxemia in in vivo mice models [45,46]. It was also found in in vivo studies that *Akkermansia muciniphila*-derived extracellular vesicles (AmEVs) levels were higher in healthy controls as compared to T2DM models and administration of AmEVs improved tight junction function and intestinal barrier integrity in an AMP-activated protein kinase (AMPK)-dependent manner [47]. In addition, administration of live or pasteurized Amuc_1100, the outer membrane protein of *Akkermansia muciniphila*, also modulates the expression of occludin and TJP1, thus, improving gut integrity [48]. Amuc_1100 also inhibits cannabinoid receptor type 1 (CB1) and toll-like receptor 2 (TLR2), thus, lowering gut permeability and systemic LPS levels. Lastly, butyrate produced by Faecalibacterium and Roseburia could also reduce gut permeability via the serotonin transporter and Peroxisome proliferator-activated receptor gamma (PPAR-γ) pathways [49]. Collectively, modulation of gut microbiota species could enhance tight junction integrity and reduce intestinal permeability through various mechanisms, including the upregulation of tight junction proteins and the production of butyrate, which could be useful in the management of T2DM.

### 3.2. Modulation of Inflammation

The pathogenesis of T2DM is also closely linked to inflammation [50]. Chronic low-grade inflammation is a significant feature of T2DM patients, with elevated inflammatory markers disrupting insulin signaling and leading to insulin resistance [51]. In vitro and in vivo studies also showed that increased levels of gut microbiota, such as Fusobacterium and Ruminococcus, can upregulate certain inflammatory cytokines, resulting in endotoxemia and inflammation [52,53,54]. Additionally, in vivo administration of *Akkermansia muciniphila* was showed to activate interleukin (IL) IL-10 in muscle, which improves glucose metabolism and has protective effects against age-related insulin resistance [48,55]. In vitro culturing with *R. intestinalis* also boosted the production of IL-22, which could restore insulin sensitivity, promote Treg cell differentiation, induce transforming growth factor beta (TGF-β), and suppress intestinal inflammation [56,57,58]. Certain species of Lactobacillus can also decrease the expression of IL-1β, IL-6, IL-8, monocyte chemoattractant protein-1 (MCP-1), intercellular adhesion molecule-1 (ICAM-1), and c-reactive protein (CRP) [59,60,61,62]. *Lactobacillus casei* inhibits interferon gamma (IFN-γ) production whereas *Roseburia intestinalis* inhibits both IFN-γ and IL-17 production [56,63,64]. Furthermore, Roseburia and Faecalibacterium produce butyrate, which inhibits the nuclear factor kappa β (*n*F-kβ) pathway [49,65,66]. Similarly, in vivo and observational studies have found that Akkermansia, Bacteroides, and Lactobacillus suppressed tumor necrosis factor alpha (TNF-α) expression while *Bacteroides thetaiotaomicron* reduced the levels of T helper cells (Th) Th1, Th2, and Th17 in mono-associated mice [56,67,68,69,70].

### 3.3. Glucose Metabolism

Impaired glucose metabolism is also another key characteristic in T2DM patients. Muscle, liver, and adipose cells become less responsive to insulin, leading to elevated blood glucose levels, while the liver continues to produce excessive glucose, further exacerbating hyperglycemia [71]. Persistent hyperglycemia damages blood vessels and nerves, increasing the risk of cardiovascular and renal complications [72]. Gut microbiota levels also affects the liver, which regulates glucose homeostasis and glucose metabolism [73]. For example, in vivo administration of the probiotic *Bifidobacterium lactis* HY8101 decreases the expression of gluconeogenesis-related genes, thus, improving the translocation of glucose transporter-4 (GLUT4) and glucose uptake [74]. *Akkermansia muciniphila* and *Lactobacillus plantarum* also suppressed expression of hepatic flavin monooxygenase 3 (Fmo3), thus, preventing the onset of hyperglycaemia and hyperlipidaemia in insulin resistant rats [48,60,75]. Lactobacillus species are also found to play a crucial role in controlling glucose metabolism. For example, in vivo administration of the probiotic *Lactobacillus casei* CCFM419 has been shown to ameliorate insulin resistance by upregulating phosphatidylinositol-3-kinase (PI3K), insulin receptor substrate 2 (IRS2), AMPK, AKT serine/threonine kinase 2 (Akt2) and glycogen synthesis in the liver [76,77]. *Lactobacillus casei* also reduced hyperglycaemia via bile acid (BA)—chloride exchange, which involves the upregulation of Chloride Channels 1 to 7 (ClC1-7), Glycine Receptor Alpha 1 (GlyRa1), Solute Carrier Family 26 Member 3 (SLC26A3), Solute Carrier Family 26 Member 6 (SLC26A6), Gamma-Aminobutyric Acid Type A Receptor Alpha 1 (GABAAa1), Bestrophin 3 (BEST3), and Cystic Fibrosis Transmembrane Conductance Regulator (CFTR) [78]. It also decreased insulin-degrading enzyme (IDE) in Caco-2 cells and insulin-like growth factor binding proteins-3 (IGFBP-3) in white adipose tissue [76,77]. Similarly, administration of the proibiotic *Lactobacillus rhamnosus* NCDC17 increased adiponectin levels in the epididymal fat while the probiotic *Lactobacillus gasseri* BNR17 boosted GLUT-4 expression in muscle, which improved insulin sensitivity [79,80]. Furthermore, some species of *Akkermansia muciniphila* and *Lactobacillii* inhibited alpha-glucosidase in vivo, which prevented the breakdown of complex carbohydrates and lowers postprandial hyperglycaemia [81]. Lastly, butyrate has been shown to act as a ligand for G protein-coupled receptors (GPCR) GPCR41 and GPCR43, thus, releasing gut hormones from entero-endocrine L-cells [82,83,84].

In addition, the gut microbiota plays an important role in BA metabolism. BA are involved in glucose metabolism as signaling molecules and cellular receptor ligands [85]. Bifidobacterium and Lactobacillus were discovered to release bile salt hydrolases to deconjugate BA, thereby enabling other gut microbiota to convert them into secondary bile acids like deoxycholic acid [86,87,88]. Secondary BAs then activate the takeda G-protein-coupled receptor 5 (TGR5) and stimulate the synthesis of glucagon-like peptide-1 (GLP-1) [89]. BA can activate the nuclear farnesoid X receptor (FXR) and membrane-bound GPCR, which decreases production of fructose-1,6-biphosphatase-1, gluconeogenic phosphoenolpyruvate carboxykinase, and glucose-6-phosphatase [90,91]. Studies on mice models also showed that knockdown of FXR lowers body weight gain and adipose tissue mass, improving glucose clearance, insulin sensitivity, and overall glucose homeostasis [92].

### 3.4. Fatty Acid Oxidation, Synthesis, and Energy Expenditure

In T2DM patients, fatty acid oxidation is frequently decreased while fatty acid synthesis increases, resulting in fat buildup in organs such as the liver and muscle, which leads to insulin resistance [93]. Furthermore, energy expenditure is often lower, promoting further weight gain and developing insulin resistance, both of which increase the risk of cardiovascular and metabolic complications [72]. Some gut microbiota have been shown to increase fatty acid oxidation, energy expenditure and reduce fatty acid synthesis [94,95]. In vivo administration of *Akkermansia muciniphila* and *Bacteroides acidifaciens* increased fatty acid oxidation in the adipose tissue [96,97,98]. *Akkermansia muciniphila* has also been found to increase the levels of 2-oleoyl glycerol (2-OG), 2-palmitoylglycerol (2-PG), and 2 acylglycerol (2-AG) while *Bacteroides acidifaciens* improved fatty acid oxidation in adipose tissue via the TGR5-PPAR-α pathway [99,100]. Administration of *Akkermansia muciniphila* and *Lactobacillus casei* have also been found to reduce serum levels of malonidialdehyde in diabetic rodents [69,101]. Butyrate, propionate, and acetate, have also been shown to decrease PPAR-γ expression, thus, increasing fatty acid oxidation [102]. In addition, butyrate also promoted thermogenesis and mitochondrial functions in muscle by inhibiting histone deacetylation in muscle [103]. Collectively, gut microbiota species can alleviate T2DM via the modulation of energy expenditure, fatty acid oxidation, and synthesis.

### 3.5. Oxidative Stress

Oxidative stress is another significant characteristic in T2DM patients. This stress is induced by an imbalance between the formation of reactive oxygen species (ROS) and antioxidant defense mechanisms, resulting in damage to insulin-sensitive organs such as the liver and pancreas, and eventually exacerbating insulin resistance, inflammation, and β-cell malfunction [104,105]. Some common oxidative markers used for T2DM diagnosis are shown in Table 1 below. Apart from antioxidant therapies such as dietary antioxidants and vitamin supplementation, modulation of gut microbiota could also be a possible intervention in reducing oxidative stress [106,107]. Recent studies have shown that Lactobacillus and Bifidobacterium species are key targets in reducing oxidative stress in T2DM patients. For instance, in vivo studies have shown that probiotic treatment with *Lactobacillus paracasei* can reduce hyperglycemia, preserve pancreatic β-cell and liver function, reduce oxidative stress, and improve dyslipidemia and inflammation [70]. Treatment with the probiotic *Lactobacillus rhamnosus* NCDC17 has also been shown to improve oral glucose tolerance test, biochemical parameters, oxidative stress, and inflammation in T2DM rat models [79]. Furthermore, administration of the probiotic *Bifidobacterium* BL21 and *Lacticaseibacillus* LRa05 significantly reduced blood glucose levels and oxidative stress in T2DM mouse models, therefore, lowering liver, cecal, and colon damage [108]. In vivo administration of the probiotics *Lactobacillus plantarum* LP104 and *Bifidobacterium longum* DD98 have also been shown to improve the production of short-chain fatty acids (SCFAs) that strengthen the gut lining, reduce lipid peroxidation and LPS translocation, thereby decreasing pro-inflammatory and oxidative responses [109,110]. In addition, other gut microbiota species like *Akkermansia muciniphila* could also improve gut barrier integrity and reduce inflammation [111,112]. Collectively, gut microbiota species can alleviate T2DM via the reduction in oxidative stress.

**Table 1:** Common oxidative markers and their relationship with gut microbiota.

Oxidative Marker	Indication in T2DM	Influence on Gut Microbiota	Reference
Advanced Glycation End-Products	Elevated levels indicate elevated oxidative stress and chronic hyperglycemia.	↑ Gut permeability ↑ Gut microbiota dysbiosis ↑ Oxidative stress	[113]
Glutathione	Decreased levels indicate diminished antioxidant defense.	↓ Gut barrier integrity ↑ Gut microbiota dysbiosis	[114]
Malondialdehyde	Elevated levels indicate oxidative damage to cell membranes.	↑ Gut permeability ↑ Inflammation ↑ Gut microbiota dysbiosis ↑ Oxidative stress	[115]
Myeloperoxidase	Elevated levels indicate inflammatory oxidative stress.	↑ Inflammation ↑ Gut microbiota dysbiosis	[116]
Nitrotyrosine	Elevated levels indicate protein damage linked to T2DM complications.	↑ Gut epithelial cell damage ↑ Inflammation ↑ Gut microbiota dysbiosis	[117]
Reactive Oxygen Species	Elevated levels indicate increased oxidative stress.	↑ Gut mucosal cell damage ↑ Growth of ROS-tolerant pathogenic strains	[118]
8-Hydroxy-2′-deoxyguanosine	Elevated levels indicate oxidative DNA damage and chronic inflammation.	↑ Gut epithelial cell damage ↑ Gut microbiota dysbiosis	[119]

Legend: ↑ Increased; ↓ Decreased.

## 4. Mechanisms of CM in the Treatment of Various Disorders via Regulating Gut Microbiota

**Figure 2:**
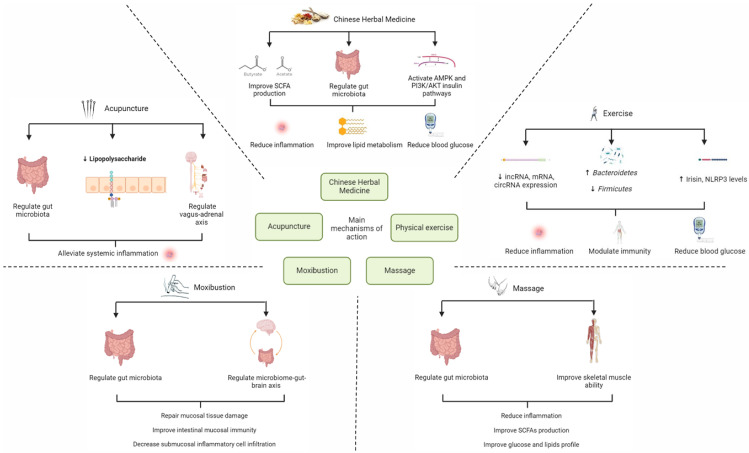
Potential mechanisms of action of various CM therapies on the modulation of gut microbiota to improve T2DM.

In CM practice, the main modes of treatment modalities can be classified into internal and external therapies. Most research examining the impact of gut microbiota modification on T2DM focuses on internal therapy, primarily including the consumption of CHM. The application of external therapies like acupuncture, moxibustion, food therapy, mind–body activities like Qigong and Tai Chi, and massage techniques like Tuina have also been the subject of other investigations [120]. In the sections below, we will review the existing evidence of these treatment modalities on alleviating T2DM. A summary diagram consolidating the mechanisms of various CM therapies on the modulation of gut microbiota to improve T2DM is shown in Figure 2 below.

### 4.1. Chinese Herbal Medicine

CHM is a key treatment modality of CM. In clinical practice, CHM is prescribed either as single herbs, or as a pre-mixed herbal concoctions [121]. A summary of studies investigating the treatment effects on T2DM via the modulation of gut microbiota by CHM are provided in Table 2 and Table 3 below.

CHMs contain a wide variety of carbohydrate and non-carbohydrate bioactive compounds. The main forms of carbohydrates found in CHM herbs include polysaccharides and oligosaccharides [122,123]. Many commonly used CHM herbs, such as *Dendrobium officinale* and *Astragalus membranaceus*, contain polysaccharides [124,125,126,127]. On the other hand, non-carbohydrate components of CHM herbs also play an important bioactive role. Some examples are polyphenols from *Dendrobium officinale*, alkaloids from *Morus alba*, and the alkaloid Berberine from *Coptis chinensis* [128,129,130]. However, the bioavailability of these components tend to decrease due to high hydrogen-bonding capacity, high molecular flexibility, and poor lipophilicity [131]. Hence, the gut microbiota plays a significant role by assisting with biotransformation of non-carbohydrate components and boosting intestinal absorption [132,133].

Among the CHMs being investigated, one prominently used CHM herb is *Coptis chinensis*. *Coptis chinensis*, is recognized as one of the most effective CHM herbs for T2DM [134]. It is utilized in many popular CHM formulas such as Ban Xia Xie Xin decoction, Xie Xin Tang decoction, Ge Gen Jiao Tai Wan granules, Ge Gen Qin Lian decoction, Huang Lian Jie Du decoction, and Shen Zhu Tiao Pi granules [135,136,137,138,139]. *Coptis chinensis* possesses multiple anti-diabetic activities. For instance, the alkaloid Berberine has been demonstrated to limit sugar digestion and absorption in the digestive tract by inhibiting disaccharide activity in a protein kinase A-dependent route and lowering sucrase-isomaltase complex mRNA expression [140]. *Coptis chinensis* has also been shown to activate the hepatic insulin-mediated signaling system and increase the compositions of Bacteroides and Clostridium, and improve BA metabolism, which could alleviate T2DM [141]. In addition, Berberine has also been demonstrated to alter gut microbiota structure by increasing goblet cell count and villi length, reversing reduced expressions of mucin, occludin, and zonula occludens-1 (ZO-1), and upregulating toll-like receptor 4 (TLR-4), NF-κB, and TNF-α [128].

**Table 2:** Alleviating T2DM Via the modulation of gut microbiota by single CHM herbs.

Herb	Part of Herb Used (If Applicable)	Type of Study	Test Subject	Total Sample Size	Main Therapeutic Effects	Key Changes in Microbiota Phylum	Reference
*Ampelopsis grossedentata*	Ethanol extract of leaves	In vivo study	ZDF rats	T2DM model T2DM model group (*n* = 6) Low-dose extract group (*n* = 6) Medium-dose extract group (*n* = 6) High-dose extract group (*n* = 6) Metformin group (*n* = 6)	Alleviate systematic inflammation Improve lipids profile Lower FBG Modulate BA production	↑ Bifidobacterium ↑ Clostridia	[142]
*Apocynum venetum*	Polysaccharide-rich extracts from leaves	In vivo study	C57BL/6 J mice	T2DM model Water extract group (*n* = 8) Ethanol extract group (*n* = 8) Saline solution group (*n* = 8)	Improve IR Improve lipids profile Lower FBG	↑ Anaeroplasma ↑ Muribaculum ↑ Odoribacter ↑ Parasutterella ↓ Aerococcus ↓ Enterococcus ↓ Klebsiella	[143]
*Astragalus membranaceus*	Polysaccharide	In vivo study	C57BL/6J mice	Non-T2DM model Non-T2DM control group (*n* = 12) T2DM model T2DM model group (*n* = 12) Mixed antibiotic group (*n* = 12) Astragalus extract group (*n* = 12) Astragalus extract and mixed antibiotic group (*n* = 12)	Alleviate systematic inflammation Improve antioxidant ability Improve IR Improve lipids profile Lower FBG	↑ Allobaculum ↑ Lactobacillus ↓ Shigella	[127]
*Coptis chinensis*	Berberine	In vivo study	ZDF rats	Non-T2DM model Impaired glucose tolerance group (*n* = 10) T2DM model Berberine group (*n* = 10) Control group (*n* = 5)	Alleviate systematic inflammation Improve IR Lower FBG Protects intestinal barrier	↑ Aggregatibacter ↑ Akkermansia ↑ Bacteroides ↑ Clostridium ↑ Eubacterium ↑ Oscillospira ↑ Roseburia ↓ Prevotella	[128]
	Water extract	In vivo study	C57BL/6 mice	Non-T2DM model Non-T2DM control group (*n* = 8) T2DM model Diabetes mellitus group (*n* = 11) Extract group (*n* = 10)	Improve IR Improve lipids profile Lower FBG Modulate BA production	↑ Bacteroides ↑ Clostridium	[141]
*Corni fructus*	Water extract from fruits	In vivo study	ICR mice	T2DM model T2DM control group (*n* = 10) Metformin group (*n* = 10) Alcohol extract group (*n* = 10) *Corni fructus* iridoidglycoside group (*n* = 10) *Corni fructus* saponin group (*n* = 10) *Corni fructus* tannin group (*n* = 10)	Alleviate systematic inflammation Improve IR Improve lipids profile Lower FBG Modulate SCFAs production	↑ Clostridium ↑ Firmicutes ↑ Lactobacillus ↓ Bacteroidetes	[144]
*Curcuma longa*	Curcumin	In vivo study	C57BLKS/J mice	T2DM model T2DM control group (*n* = 10) Control and curcumin group (*n* = 10) Dextran sodium sulfate group (*n* = 14) Dextran sodium sulfate and curcumin group (*n* = 13)	Lower FBG Improve immune regulation Improve IR	↑ Candidatus ↑ Eubacterium	[145]
	Tetrahydrocurcumin	In vivo study	SPF mice	T2DM model Model group (*n* = 7) Low-dose group (*n* = 7) High-dose group (*n* = 7)	Improve IR Lower FBG	↑ Bacteroidetes ↑ Firmicutes ↓ Actinobacteria ↓ Proteobacteria	[146]
*Dendrobium officinale*	*Dendrobium officinale* supplement	In vivo study	BKS.Cg-Dock7m +/+Leprdb/Nju mice	T2DM model Placebo group (*n* = 6) *Dendrobium officinale* supplement group (*n* = 6)	Lower FBG	↑ Akkermansia ↑ Clostridium ↑ Flavonifractor ↑ Parabacteroides	[124]
	Polyphenol extract	In vivo study	BKS-db mice	Non-T2DM model Non-T2DM control group (*n* = 8) T2DM model T2DM group (*n* = 8) Metformin group (*n* = 8) Low-dose group (*n* = 8) Medium-dose group (*n* = 8) High-dose group (*n* = 8)	Alleviate systematic inflammation Improve antioxidant ability Improve IR Improve lipids profile Lower FBG	↑ Akkermansia ↑ Bacteroidetes ↓ Escherichia	[129]
*Edgeworthia gardneri*	Water extract	In vivo study	C57BL/6J mice	Non-T2DM model Non-T2DM control group (*n* = 8) T2DM model T2DM group (*n* = 8) Metformin group (*n* = 8) Low-dose group (*n* = 8) Medium-dose group (*n* = 8) High-dose group (*n* = 8)	Alleviate systematic inflammation Improve antioxidant ability Improve IR Improve lipids profile Lower FBG Modulate SCFAs production	↑ Bacteroidetes ↑ Clostridiales ↓ Deferribacteres ↓ Dorea ↓ Firmicutes ↓ Lachnospiraceae ↓ Proteobacteria ↓ Rikenellaceae	[147]
*Ganoderma atrum*	Polysaccharide	In vivo study	SD rats	Non-T2DM model Non-T2DM control group (*n* = 8) T2DM model T2DM group (*n* = 8) *Ganoderma atrum* polysaccharide group (*n* = 8)	Alleviate systematic inflammation Improve antioxidant ability Improve IR Improve lipids profile Lower body weight Lower FBG Modulate SCFAs production	↑ Blautia ↑ Bacteroides ↑ Dehalobacterium ↑ Parabacteroides ↓ Aerococcus ↓ Corynebactrium ↓ Proteus ↓ Ruminococcus	[148]
	Polysaccharide F31	In vivo study	KM mice	Non-T2DM model Non-T2DM control group (*n* = 8) T2DM model T2DM group (*n* = 8) Low-dose group (*n* = 8) High-dose group (*n* = 8)	Alleviate systematic inflammation Improve antioxidant ability Improve IR Lower FBG	↑ Bacteroides ↑ Bacteroidetes ↑ Lactobacillus ↑ Ruminococcaceae ↓ Firmicutes	[126]
*Gastrodia elata*	Water extract	In vivo study	C57BL/6 mice	Not reported	Alleviate systematic inflammation Improve lipids profile Lower FBG Modulate BA production	↑ Faecalibaculum ↑ Lactobacillus ↑ Mucispirillum	[149]
*Hypericum attenuatum*	Whole plant extract	In vivo study	KM mice	Non-T2DM model Non-T2DM control group (*n* = 10) T2DM model T2DM group (*n* = 10) Metformin group (*n* = 10) Low-dose group (*n* = 10) Medium-dose group (*n* = 10) High-dose group (*n* = 10)	Improve IR Improve lipids profile Lower FBG Modulate SCFAs production	↑ Firmicutes ↓ Bacteroidetes ↓ Proteobacteria	[150]
*Inonotus obliquus*	Polysaccharide	In vivo study	KM mice	Non-T2DM model Non-T2DM control group (*n* = 8) T2DM model T2DM group (*n* = 8) Metformin group (*n* = 8) Low-dose group (*n* = 8) Medium-dose group (*n* = 8) High-dose group (*n* = 8)	Improve IR Improve lipids profile Lower FBG Protects intestinal barrier	↑ Bacteroidetes	[151]
*Lycium barbarum*	Water extract from leaves	In vivo study	SPF-grade rats	Non-T2DM model Non-T2DM control group (*n* = 8) T2DM model T2DM group (*n* = 8) Metformin group (*n* = 8) Low-dose group (*n* = 8) High-dose group (*n* = 8)	Improve IR Improve lipids profile Lower FBG	↓ Blautia ↓ Coprococcus ↓ Marvinbryantia ↓ Parasutterella ↓ Prevotellaceae ↓ Ruminococcus	[152]
*Maydis stigma*	Polysaccharide	In vivo study	KM mice	Non-T2DM model Non-T2DM control group (*n* = 8) T2DM model T2DM group (*n* = 8) Dimethylbiguanide group (*n* = 8) Low-dose group (*n* = 8) Medium-dose group (*n* = 8) High-dose group (*n* = 8)	Lower FBG	↑ Bacteroidetes ↑ Lactobacillus	[153]
*Momordica charantia*	Polysaccharide	In vivo study	Wistar rats	Non-T2DM model Control group (*n* = 10) Medium-dose fermented polysaccharide group (*n* = 10) Unfermented polysaccharide group (*n* = 10) T2DM model Control group (*n* = 10) Low-dose fermented polysaccharide group (*n* = 10) Medium-dose fermented polysaccharide group (*n* = 10) High-dose fermented polysaccharide group (*n* = 10) Unfermented polysaccharide group (*n* = 10)	Improve antioxidant ability Improve IR Improve lipids profile Lower FBG Modulate SCFAs production	↑ Lactococcus ↑ Prevotella	[154]
*Morus alba*	Leaf powder	In vivo study	SD rats	T2DM model Vehicle control group (*n* = 6) Treatment group (*n* = 6) Positive control group (*n* = 6) Negative control group (*n* = 6)	Improve IR Improve lipids profile Lower FBG Modulate SCFAs production	↑ Bacteroidetes ↑ Clostridia ↑ Proteobacteria	[155]
	Ethanol extract from leaves	In vivo study	SD rats	Non-T2DM model Non-T2DM control group (*n* = 6) T2DM model T2DM group (*n* = 6) Treatment group (*n* = 6)	Improve lipids profile Lower FBG	↑ Bacteroidetes ↑ Firmicutes ↓ Actinobacteria ↓ Bifidobacterium	[156]
	Alkaloids from the twig	In vivo study	KK-Ay mice	T2DM model T2DM group (*n* = 8) Low-dose group (*n* = 8) High-dose group (*n* = 8)	Alleviate systematic inflammation Improve IR Improve lipids profile Lower FBG Protects intestinal barrier	↑ Bacteroidaceae ↑ Verrucomicrobia ↓ Desulfovibrionaceae ↓ Rikenellaceae	[130]
	Polysaccharide	In vivo study	db/db mice	T2DM model T2DM group (*n* = 10) Metformin group (*n* = 10) Low dose group (*n* = 10) Medium dose group (*n* = 10) High dose group (*n* = 10)	Improve antioxidant ability Improve lipids profile Improved oral glucose tolerance Lower FBG	↑ Allobaculum ↑ Akkermansia ↑ Bacteroidales ↑ Bacteroides ↑ Lactobacillus ↓ Enterococcus ↓ Staphylococcus	[157]
*Panax ginseng*	Ginsenoside Rb1	In vivo study	Kkay mice	T2DM model T2DM group without antibiotic treatment (*n* = 10) Metformin group without antibiotic treatment (*n* = 10) Ginsenoside group without antibiotic treatment (*n* = 10) T2DM group with antibiotic treatment (*n* = 10) Metformin group with antibiotic treatment (*n* = 10) Ginsenoside group with antibiotic treatment (*n* = 10)	Improve IR Improve lipids profile Lower FBG Protects intestinal barrier	↑ Parasutterella ↓ Alistipes ↓ Anaeroplasma ↓ Odoribacter ↓ Prevotellaceae_	[158]
	Ginsenoside Rg1	In vivo study	Wistar rats	Non-T2DM model Non-T2DM control group (*n* = 8) T2DM model T2DM group (*n* = 8) Metformin group (*n* = 8) Low dose group (*n* = 8) High dose group (*n* = 8)		↑ Lachnospiraceae ↑ Romboutsia ↑ Roseburia	[159]
	Ginsenoside Rg5	In vivo study		Non- T2DM model Non-T2DM control group (*n* = 7) Non-T2DM antibiotic control group (*n* = 7) T2DM model T2DM vehicle treatment group (*n* = 7) Rg5 vehicle treatment group (*n* = 7) Antibiotic vehicle treatment group (*n* = 7) Rg5 non = vehicle treatment group (*n* = 7)		↑ Bacteroidetes ↑ Proteobacteria ↓ Firmicutes ↓ Verrucomicrobia	[160]
*Physalis alkekengi var. francheti*	Polysaccharide	In vivo study	KM mice	Non-T2DM model Non-T2DM control group (*n* = 10) T2DM model T2DM group (*n* = 10) Dimethybiguanide group (*n* = 10) Low dose group (*n* = 10) High dose group (*n* = 10)	Alleviate systematic inflammation Improve lipids profile Lower FBG	↑ Bacteroides ↑ Clostridium ↑ Lactobacillus ↓ Enterobacter	[161]
*Plantago asiatica*	Polysaccharide from the seeds	In vivo study	Wistar rats	Non-T2DM model Non-T2DM control group (*n* = 10) Non-T2DM medium dose group (*n* = 10) T2DM model T2DM control group (*n* = 10) Metformin group (*n* = 10) Low dose group (*n* = 10) Medium dose group (*n*-10) High dose group (*n* = 10)	Improve antioxidant ability Improve IR Improve lipids profile Lower FBG Modulate SCFAs production	↑ Bacteroides ↑ Lactobacillus ↑ Prevotella ↓ Alistipes	[125]
*Sanghuangporus vaninii*	Fruit body polysaccharide extract	In vivo study	ICR mice	Non-T2DM model Non-T2DM control group (*n* = 8) T2DM model T2DM group (*n* = 8) Metformin group (*n* = 8) Low-dose group (*n* = 8) High-dose group (*n* = 8)	Improve IR Improve lipids profile Lower FBG Modulate SCFAs production	↑ Alloprevotella ↑ Dubosiella ↑ Weissella ↓ Flavonifractor ↓ Lactobacillus ↓ Odoribacter	[162]
*Tribulus terrestris*	Ethanol extract	In vivo study	SD rats	Not reported	Improve lipids profile Lower FBG Modulate BA production Modulate SCFAs production	↑ Bacteroidetes ↑ Bifidobacterium ↓ Firmicutes	[163]

Abbreviations: Fasting blood glucose (FBG), Insulin resistance (IR), Short chain fatty acids (SCFA), Bile acids (BA). Legend: ↑ Increased abundance; ↓ Decreased abundance.

**Table 3:** Alleviating T2DM Via the modulation of gut microbiota by CHM formulas.

Herbal Formula	Herbal Formula Composition	Type of Study	Test Subject	Total Sample Size	Main Therapeutic Effects	Key Changes in Microbiota Phylum	Reference
AMC	*Aloe vera* *Coptis chinensis* *Momordica charantia* Red yeast rice *Rhizoma anemarrhenae* *Salvia miltiorrhiza* *Schisandra chinensis* *Zingiber officinale*	Randomized controlled trial	T2DM patients	T2DM model Metformin group (*n* = 100) AMC treatment group (*n* = 100)	Improve IR Lower FBG	↑ Blautia ↑ Coprococcus ↑ Faecalibacterium ↑ Gemmiger ↑ Megamonas ↑ Roseburia	[164]
Bai Hu Ren Sheng decoction	*Glycyrrhiza uralensis* *Gypsum* *Japonica rice* *Panax ginseng* *Rhizoma anemarrhenae*	In vivo study	SD rats	Non-T2DM model Non-T2DM control group (*n* = 10) T2DM model T2DM group (*n* = 10) Metformin group (*n* = 10) Low dose group (*n* = 10) High dose group (*n* = 10)	Alleviate systemic inflammation Improve antioxidant ability Improve IR Improve lipid metabolism Lower FBG	↑ Anaerostipes ↑ Blautia ↑ Lactobacillus ↓ Allobaculum ↓ Candidatus ↓ Ruminococcus ↓ Saccharimonas	[165]
Bu Yang Huan Wu decoction	*Angelicae sinensis* *Astragalus membranaceus* *Carthami flos* *Ligusticum striatum* *Paeoniae rubra* *Persicae semen* *Pheretima*	In vivo study	ZDF rats and ZLC rats	Non-T2DM model Non-T2DM control group (*n* = 6) T2DM model T2DM group (*n* = 6) Metformin group (*n* = 6) Bu Yang Huan Wu decoction group (*n* = 6)	Improve lipid metabolism Lower FBG	↑ Bacteroidetes ↑ Blautia. ↑ Lactobacillus ↓ Firmicutes	[166]
Dang Gui Bu Xue decoction	*Astragalus membranaceus* *Angelicae sinensis*	In vivo study	Goto Kakizaki (GK) rats	Non-T2DM model Non-T2DM control group (*n* = 6) T2DM model T2DM group (*n* = 6) Dang Gui Bu Xue decoction group (*n* = 6)	Alleviate systemic inflammation Improve antioxidant ability Improve IR Improve lipid metabolism Lower FBG	↑ Adlercreutzia ↑ Peptostreptococcaceae ↑ Oscillospiraceae ↓ Firmicutes	[167]
Ge Gen Jiao Tai Wan formula	*Coptis chinensis* *Cortex cinnamomi* *Pueraria lobata*	In vivo study	SD rats	Non-T2DM model Non-T2DM control group (*n* = 10) T2DM model T2DM group (*n* = 10) Ge Gen Jiao Tai Wan formula group (*n* = 10) Fecal transplant group (*n* = 10) Metformin group (*n* = 7) Antibiotics group (*n* = 10) Ge Gen Jiao Tai Wan formula and antibiotics group (*n* = 10)	Improve IR Improve lipid metabolism Lower FBG	↑ Firmicutes ↑ Lactobacillus	[138]
Ge Gen Qin Lian decoction	*Coptis chinensis* *Glycyrrhiza uralensis* *Pueraria lobata* *Scutellaria baicalensis*	Randomized controlled trial	T2DM patients	T2DM model Placebo group (*n* = 56) Low dose group (*n* = 56) Moderate dose group (*n* = 56) High dose group (*n* = 56)	Alleviate systematic inflammation Lower FBG	↑ Bifidobacterium ↑ Faecalibacterium ↑ Gemmiger ↓ Alistipes ↓ Parabacteroides ↓ Pseudobutyrivibrio	[168]
		In vivo study	Wistar rats	Non-T2DM model Non-T2DM control group (*n* = 6) T2DM model T2DM group (*n* = 6) Metformin group (*n* = 6) Ge Gen Qin Lian decoction group (*n* = 6)	Alleviate systematic inflammation Improve IR Improve lipid metabolism Lower FBG Protects intestinal barrier	↑ Acetatifactor ↑ Flavonifractor ↓ Anaerofustis ↓ Butyricicoccus ↓ Butyricimonas ↓ Gammaproteobacteria	[169]
Huang Lian Jie Du decoction	*Coptis chinensis* *Gardeniae Fructus* *Phellodendri Cortex* *Scutellaria baicalensis*	In vivo study	SD rats	Non-T2DM model Non-T2DM control group (*n* = 8) T2DM model T2DM group (*n* = 8) Huang Lian Jie Du decoction group (*n* = 8)	Alleviate systematic inflammation Improve antioxidant ability Improved IR Improve lipid metabolism Lower FBG	↑ Akkermansia ↑ Blautia ↑ Parabacteroides ↓ Aerococcus ↓ Staphylococcus	[170]
Jiang Tang Jing granules	*Astragalus membranaceus* *Coicis semen* *Crataegi fructus* *Dioscorea oppositifolia* *Hirudo* *Polygonati rhizoma* *Pueraria lobata* *Semen brassicae*	In vivo study	SD rats	Non-T2DM model Non-T2DM control group (*n* = 6) T2DM model T2DM group (*n* = 6) Linagliptin group (*n* = 6) Huang Lian Jie Du decoction group (*n* = 6)	Improve IR Lower FBG	↑ Bacteroides ↓ Actinobacteria	[171]
Jiang Tang San Huang pill	*Astragalus membranaceus* *Cinnamomum cassia* *Glycyrrhiza uralensis* *Ophiopogon japonicus* *Persicae semen* *Rehmannia glutinosa* *Rheum palmatum* *Scrophularia ningpoensis*	In vivo study	SD rats	Non-T2DM model Non-T2DM control group (*n* = 10) T2DM model T2DM group (*n* = 10) Metformin group (*n* = 10) Low dose group (*n* = 10) Medium dose group (*n* = 10) High dose group (*n* = 10)	Alleviate systemic inflammation Improve IR Improve lipid metabolism Lower FBG	↑ Bacteroides ↑ Bifidobacterium ↑ Clostridium ↑ Lactobacillus	[172]
Jin Qi Jiang Tang tablets	*Astragalus membranaceus* *Coptis chinensis* *Lonicera japonica*	In vivo study	C57BL/6J mice	Non-T2DM model Non-T2DM control group (*n* = 5) T2DM model T2DM group (*n* = 5) Low dose group (*n* = 5) High dose group (*n* = 5)	Alleviate systemic inflammation Improve IR Lower FBG Protects intestinal barrier	↑ Akkermansia ↓ Desulfovibrio	[173]
Liu Wei Di Huang pills	*Corni fructus* *Cortex moutan* *Dioscorea oppositifolia* *Rehmannia glutinosa* *Rhizoma alismatis* *Poria cocos*	In vivo study	Goto Kakizaki (GK) rats	Non-T2DM model Non-T2DM control group (*n* = 6) T2DM model T2DM group (*n* = 6) Metformin group (*n* = 6) Liu Wei Di Huang pills group (*n* = 6)	Improve IR Improve lipid metabolism Lower FBG Modulate SCFAs production	↑ Allobaculum ↑ Firmicutes ↑ Lactobacillus ↑ Ruminococcus	[174]
LLKL formula	*Crocus sativus* *Edgeworthia gardneri* *Sibiraea angustata*	In vivo study	ZDF rats	Non-T2DM model Non-T2DM control group (*n* = 8) T2DM model T2DM group (*n* = 8) Metformin group (*n* = 8) LLKL low-dose group (*n* = 8) LLKL medium-dose group (*n* = 8) LLKL high-dose group (*n* = 8)	Alleviate systemic inflammation Improve IR Improve lipid metabolism Lower FBG	↑ Bacteroidetes ↑ Proteobacteria ↓ Firmicutes	[175]
Pi Dan Jian Qing decoction	*Astragalus membranaceus* *Coptis chinensis* *Potentilla discolor* *Pseudostellaria heterophylla* *Pueraria lobata* *Rhizoma atractylodis* *Salvia miltiorrhiza* *Scrophularia ningpoensis* *Scutellaria baicalensis*	Randomized controlled trial	T2DM patients	T2DM model Control group (*n* = 32) Pi Dan Jian Qing decoction group (*n* = −35)	Alleviate systemic inflammation Improve antioxidant ability Improve IR Improve lipid metabolism Lower FBG	↑ Akkermansia ↑ Bacteroides ↑ Blautia ↑ Desulfovibrio ↑ Lactobacillus ↓ Prevotella	[176]
Qi Jian mixture	*Astragalus membranaceus* *Coptis chinensis* *Pueraria lobata* *Ramulus euonymi*	In vivo study	KKay mice	Non-T2DM model Non-T2DM control group (*n* = 6) T2DM model T2DM group (*n* = 6) Metformin group (*n* = 6) Qi Jian mixture low dose group (*n* = 6) Qi Jian mixture high dose group (*n* = 6) Ge Gen Qin Lian decoction group (*n* = 6)	Alleviate systematic inflammation Improved IR Improve lipid metabolism Lower FBG	↑ Bacteroides	[177]
Shen Lian decoction	*Coptis chinensis* *Panax ginseng*	In vivo study	C57BL/KsJ-db/db mice	Non-T2DM model Non-T2DM control group (*n* = 8) T2DM model T2DM group (*n* = 8) Metformin group (*n* = 8) *Coptis chinensis* group (*n* = 8) *Panax ginseng* group (*n* = 8) Shen Lian decoction group (*n* = 8)	Improve IR Lower FBG	↑ Bacteroidaceae ↓ Helicobacteraceae ↓ Prevotellaceae ↓ Rikenellaceae	[178]
Shen Qi compound	*Astragalus membranaceus* *Corni fructus* *Dioscorea oppositifolia* *Panax ginseng* *Rehmannia glutinosa* *Rheum palmatum* *Salvia miltiorrhiza* *Trichosanthes kirilowii*	In vivo study	Goto Kakizaki (GK) rats	Non-T2DM model Non-T2DM control group (*n* = 10) T2DM model T2DM group (*n* = 10) Sitagliptin group (*n* = 10) Shen Qi compound group (*n* = 10)	Alleviate systemic inflammation Lower FBG Modulate SCFAs production Improve IR Improve lipid metabolism Protects intestinal barrier	↑ Bacteroides ↑ Blautia ↑ Butyricimonas ↑ Prevotellaceae ↑ Roseburia ↓ Lactobacillus ↓ Rothia	[179]
Shen Zhu Tiao Pi granule	*Codonopsis pilosula* *Coptis chinensis* *Pericarpium Citri Reticulatae* *Poria cocos* *Pueraria lobata* *Rhizoma atractylodis,* *Rhizoma pinelliae*	In vivo study	Goto-Kakizaki rats and Wistar rats	Non-T2DM model Non-T2DM control group (*n* = 6) T2DM model T2DM group (*n* = 6) Acarbose group (*n* = 6) Shen Zhu Tiao Pi granule group (*n* = 6)	Improve lipid metabolism Lower FBG	↑ Lactobacillus ↓ Allobaculum ↓ Bacteroidetes ↓ Desulfovibrionaceae	[136]
Xie Xin Tang decoction	*Coptis chinensis* *Rhizoma Rhei* *Scutellaria baicalensis*	In vivo study	SD rats	Non-T2DM model Non-T2DM control group (*n* = 6) T2DM model T2DM group (*n* = 6) Xie Xin Tang decoction group (*n* = 6)	Alleviate systematic inflammation Improve lipid metabolism Lower FBG	↑ Alloprevotella ↑ Barnesiella ↑ Eubacterium ↑ Lachnospiraceae ↑ Papillibacter ↑ Prevotellaceae ↓ Adlercreutzia ↓ Blautia	[139]

Abbreviations: Fasting blood glucose (FBG), Insulin resistance (IR), Short chain fatty acids (SCFA), Bile acids (BA). Legend: ↑ Increased abundance; ↓ Decreased abundance.

### 4.2. Acupuncture

Acupuncture is a key treatment modality of CM practiced extensively in China for over 4000 years [180]. Some acupuncture techniques include manual acupuncture, electroacupuncture, and auricular acupuncture, which involve inserting and manipulating small needles into precise sites on the body to produce therapeutic results [181,182]. Studies have also shown acupuncture sites to align with cutaneous areas with strong electrical conductivity and unique histologic alterations [183]. Due to its potential in treatment, acupuncture was recommended in China’s guidelines for T2DM treatment in 2020 [184]. A summary of studies investigating the treatment effects on T2DM by acupuncture is provided in Table 4 below.

In addition to lowering fasting blood glucose (FBG) levels, the studies below have also shown that acupuncture possesses other useful therapeutic effects such as improving insulin resistance, lipid metabolism, and managing inflammation. Although the precise mechanisms are unclear at this juncture, some theories have been postulated. Recent studies have proposed that impaired parasympathetic function is a key pathological feature of T2DM [185]. The VN is part of the parasympathetic nervous system, and it is made up of afferent and efferent fibers [186]. The VN detects microbiota metabolites through its afferent fibers and transmits the information to the central nervous system, resulting in a cholinergic anti-inflammatory pathway that decreases peripheral inflammation and intestinal permeability [187]. Collectively, acupuncture drives the vagal–adrenal axis and reduces inflammation, which could contribute to the management of T2DM [188,189]. Furthermore, it is hypothesized that electroacupuncture could excite the somatic afferent fibers of the VN to improve insulin sensitivity [190]. Another possible mechanism is by lowering free fatty acids and improving the recovery of IRS1 and GLUT4 [191]. Xu et al. also showed that high frequency electroacupuncture stimulated distal colonic transit, which may be due to downregulation of apoptosis and proliferation of interstitial cells of Cajal [192]. Further studies then showed that electroacupuncture could regulate the IKKβ/NF-κB-JNK–IRS-1–AKT pathway, thus, contributing to increased tight junction protein expression and reduced inflammatory factors [193]. Collectively, it is, thus, postulated that electroacupuncture increased the diversity of gut flora, promotes colonic motility, and contributes to its hypoglycemic effect.

At this juncture, only a few studies have attempted to elucidate the mechanism of acupuncture in T2DM management. Hence, this is a potential area of research, which should be considered in the future.

**Table 4:** Alleviating T2DM via the modulation of gut microbiota by acupuncture.

Type of Acupuncture	Acupoints Selected	Type of Study	Test Subject	Total Sample Size	Main Therapeutic Effects	Key Changes in Microbiota Phylum	Reference
Electroacupuncture	Bilateral ST36	In vivo study	BKS.Cg m+/+ Leprdbrdb/J (db/db) diabetic mice	Non-T2DM model Non-T2DM control group (*n* = 8) T2DM model T2DM group (*n* = 8) Electroacupuncture treatment group (*n* = 8)	Improve IR Improve lipid metabolism Lower FBG	↑ Lactobacillus ↓ Bacteroides ↓ Clostridia ↓ Lachnospiraceae ↓ Ruminococcaceae	[194]
		In vivo study	C57BL/6 mice and Kit^W/Wv^ mice	Non-T2DM model Non-T2DM control group (*n* = 10) High fat diet group (*n* = 10) T2DM model T2DM group (*n* = 10) Electroacupuncture treatment group (*n* = 10) Sham electroacupuncture treatment group (*n* = 10)	Alleviate systematic inflammation Improve IR Lower FBG	↓ Desulfovibrio ↓ Firmicutes ↓ Lachnoclostridium ↓ Lachnospiraceae ↓ Odoribacter ↓ Oscillibacter	[193]
	Bilateral BL13, BL20, BL23, LI4, LR3, ST36, and SP6	In vivo study	SPF-grade rats	Non-T2DM model Non-T2DM control group (*n* = 8) T2DM model T2DM group (*n* = 8) Electroacupuncture treatment group (*n* = 8)	Alleviate systematic inflammation Improve IR Improve lipids profile Lower FBG Modulate SCFAs production	↑ Blautia ↑ Lactobacillus ↓ Alistipes ↓ Helicobacter ↓ Prevotella	[195]
		In vivo study		Non-T2DM model Non-T2DM control group (*n* = 8) T2DM model T2DM group (*n* = 8) Metformin group (*n* = 8) Electroacupuncture treatment group (*n* = 8)	Improve IR Improve lipids profile Lower FBG Modulate BA production	↑ Actinobacteria ↑ Firmicutes	[196]
	Bilateral ST36 and RN12	In vivo study	SPF-grade rats	Non-T2DM model Non-T2DM control group (*n* = 8) T2DM model T2DM group (*n* = 8) Metformin group (*n* = 8) Electroacupuncture treatment group (*n* = 8)	Alleviate systematic inflammation Improve lipids profile Lower FBG	↑ Firmicutes ↓ Bacteroides ↓ Eubacterium	[197]

Abbreviations: Fasting blood glucose (FBG), Insulin resistance (IR), Short chain fatty acids (SCFA), Bile acids (BA). Legend: ↑ Increased abundance; ↓ Decreased abundance.

### 4.3. Moxibustion

Moxibustion is another key treatment modality of CM, which involves using moxa as burning material [198]. Moxa is a cotton wool-like material made from the leaves of *Artemisia vulgaris*, which possesses anti-inflammatory, hepatoprotective, antioxidant, and anti-tumoral properties [199]. Although no studies have directly evaluated the effect of moxibustion on gut microbiota modulation and T2DM improvement, the thermal effect of moxibustion has proven to be effective in treating other similar conditions. For instance, the thermal effect of moxibustion can repair mucosal tissue damage, improve intestinal mucosal immunity, and decrease submucosal inflammatory cell infiltration [200]. Moxibustion has also been proposed to target the microbiome–gut–brain axis, hence, reducing inflammation caused by the neuroendocrine-immune system [201]. Moxibustion treatment at bilateral ST25 acupoint has also been shown to reduce Proteobacteria, Saccharibacteria, Sphingomonas, and Barnesiella significantly in patients with inflammatory bowel disease [202]. In patients with intestinal mucositis, the results indicated that mild moxibustion at bilateral ST 25 helped to re-establish the α and β diversity of gut microbiota by increasing Lactobacillus, Roseburia, and Escherichia, thus, alleviating mucosal damage and inflammation [203]. Considering the presence of existing evidence showing that moxibustion can modulate gut microbiota in diseases with similar pathological factors as T2DM, investigating moxibustion and its potential effects in improving T2DM via gut microbiota modulation is also a potential area of research that should be considered in the future.

### 4.4. Massage

Tuina, a traditional form of hands-on manipulation treatment that combines modern scientific understanding with traditional practice, is the primary form of massage therapy in CM [204]. Furthermore, Tuina is a safe, effective, cost-efficient, and non-invasive intervention method that is well accepted by many [205,206]. Although CM-specific evidence investigating the effects of Tuina is currently lacking, studies conducted on other forms of massage have shown that it can affect gut microbiota composition and alleviate T2DM. For instance, Xie et al. found that abdominal massage guided by CM Tuina principles improved blood glucose and lipid metabolism by increasing Bifidobacteria and Lactobacillus while decreasing Enterococcus and Enterobacter [207]. Another study on patients with chronic functional constipation found that acupoint massage therapy could increase the amount of *Pseudobutyrivibrio*, a butyric acid-producing genus that produces short chain fatty acids (SCFAs) to reduce inflammation via GPCRs [208]. Therefore, future studies could build on these positive findings and focus on investigating Tuina therapy to validate its effects on gut microbiota modulation in treating T2DM.

### 4.5. Chinese Medicine-Guided Physical Exercise

Chinese Medicine-guided physical exercise serves as a key treatment modality of CM, which consists of CM exercises such as Taichi and Qigong [209]. Although CM-specific evidence on T2DM treatment is presently limited, studies conducted on other forms of CM exercise have shown that exercise can affect gut microbiota composition. Past systematic reviews have shown positive results that show CM exercises such as Taichi, Qigong, and Baduanjin improve blood glucose and lipid levels, thus, showing great potential in treatment for T2DM [210,211,212,213]. However, how these CM exercises regulate gut microbiota in T2DM patients is not well examined. In other studies, it was found that moderate intensity activity improved immune function, oxidative stress, and inflammation [214,215]. In a study conducted on patients with myalgic encephalomyelitis, it was found that maximal exercise challenge increased Bacteroidetes and decreased the composition of Firmicutes [216]. This finding has been backed by both studies on animal and human subjects, which showed improvements caused by modulating the gut microbiota [217,218,219,220,221,222]. This could be essential as reducing the proportion of Firmicutes and increasing population of *Bacteroides* has been associated with alleviating T2DM [223]. From an immunological standpoint, the putative hypoglycemic mechanism of CM exercises in prediabetes patients was proposed to relate to increased irisin and suppression of the nucleotide-binding-domain, leucine-rich repeat containing the protein 3 (*n*LRP3) inflammatory pathway [224]. CM exercise was also discovered to modulate aberrant lncRNA, mRNA, and circRNA expression, which improved T2DM patients’ depressive symptoms and blood glucose levels [225]. Given the positive findings in CM exercise-related animal and human studies, future clinical trials could be designed with CM exercises such as Taichi and Qigong as the main mode of intervention to validate CM exercises in treating T2DM.

## 5. Conclusions

Our present study summarized the role of gut microbiota in T2DM pathogenesis, provided an updated review of CHM, and it also evaluated other CM treatment modalities for T2DM. Clinical research has also shown that CM therapies that modify the gut microbiota can improve T2DM. Some of the key mechanisms of action mentioned above include enhancing gut barrier function, lowering systemic inflammation, improving glycemic management, and decreasing insulin resistance. Therefore, CM is a viable treatment option for T2DM, and future studies should attempt to evaluate the feasibility of larger-scale implementation of CM in T2DM treatment. Lastly, we would like to address some gaps in research and offer some perspectives and directions for future research below.

Firstly, we observed that the bulk of investigations are conducted in vivo using mouse and rat models. Although the findings from these studies are positive, more work could be performed to better understand the underlying mechanisms of action of gut microbiota modulation in T2DM treatment. Future in vivo studies could investigate the underlying mechanisms linking changes in specific gut microbiota species to therapeutic effects. Given that many of these microbiota species are naturally present in the body, it is essential to analyze dose–response relationships and to determine the optimal concentration, delivery method, and administration protocol for each microbiota species to maintain microbial balance. Additionally, future research should also include toxicological studies to evaluate treatment safety. As most studies reviewed are in vivo, clinical trials are necessary to confirm the safety of these treatments before they can be integrated into clinical practice. Therefore, future studies could attempt to conduct high-quality and large-scale clinical trials to translate these promising findings from animal models to humans.

Secondly, in terms of gut microbiota, we observed that most studies analyzed stool samples. Although conducting biopsies would be invasive for patients and not suitable for healthy controls, past research has noted that the stool samples might not fully reflect the actual gut microbiota composition [226,227]. Hence, future research could focus on developing less invasive approaches to obtain samples from different sites of the intestine to improve overall representation. Another point of contention raised by other groups today is that gut microbiota should be classified according to function rather than taxonomic similarities and that function-based analysis should be investigated [228,229]. Therefore, novel bioinformatic techniques could also be utilized to elucidate meaningful correlations between microbial composition and host physiological processes [230,231]. Finally, to build on the findings of animal studies, well-defined gnotobiotic models such as humanized gut microbiota could be utilized [232].

Lastly, we propose that future CM-related research could focus on the following areas. In terms of research on CHM, more research could also be performed to elucidate the bioavailability, pharmacokinetics, and metabolic pathways of the various CHMs and its effect on gut microbiota modulation. Furthermore, the optimal beneficial dosage of bioactive components could be better elucidated in future studies. Among the CHM herbs reviewed in our study, we also observed that many of them are currently classified as “Medicine and Food Homology” (MFH) herbs by the Chinese Ministry of Health [233]. Because of their flexibility and safety in use, MFH medicinal foods are important for chronic conditions like T2DM that require longer periods of consumption as they can be integrated into one’s daily diet [234]. For instance, a food item representing the concept of “Medicine and Food Homology” is Poria cake, which was created to improve one’s appetite and digestive functions [235,236]. Due to its ease of incorporation into one’s diet, it could be a long-term therapy for treating T2DM, supplementing existing medications that the T2DM patient is consuming. In terms of the types of CM interventions currently investigated, we also observed that CHM and acupuncture are more thoroughly investigated than other CM treatment modalities. Other CM treatments like moxibustion, massage, and CM exercises have been widely examined, although their use in the treatment of T2DM and gut microbiota regulation have not been investigated. Hence, future research could further investigate the specific impacts of these interventions on gut microbiota regulation and T2DM treatment.

In closing, CM therapies are useful in modulating gut microbiota and improving T2DM management. While current research suggests that CM may be a promising alternative for managing T2DM, future studies could focus on several key areas. First, improved in vivo study designs are needed to investigate the biomolecular mechanisms, optimal dosages, and safety of various CM interventions. Translating these findings to human models will be essential before clinical integration. Secondly, future studies could also focus on developing less invasive sampling methods for comprehensive gut microbiota analysis. Thirdly, future studies could investigate other CM interventions, including moxibustion and CM exercises, and explore other CM interventions, such as moxibustion and CM exercises, to assess their effects on gut microbiota and T2DM management. Finally, future research could focus on incorporating CHMs into food and pharmaceuticals for T2DM.

## Data Availability

The original contributions presented in this study are included in the article. Further inquiries can be directed to the corresponding authors.
